# Placental Shear Wave Elastography Assessment in Early and Late Fetal Growth Restriction

**DOI:** 10.3390/jcm14144980

**Published:** 2025-07-14

**Authors:** Erika Cavanagh, Kylie Crawford, Jesrine Hong, Davide Fontanarosa, Christopher Edwards, Marie-Luise Wille, Jennifer Hong, Vicki L. Clifton, Sailesh Kumar

**Affiliations:** 1 Mater Research Institute, University of Queensland, Level 3, Aubigny Place, Raymond Terrace, South Brisbane, QLD 4101, Australia; ej1.robinson@hdr.qut.edu.au (E.C.); jesrine.hong@uq.edu.au (J.H.);; 2 School of Clinical Sciences, Faculty of Health, Queensland University of Technology, Brisbane, QLD 4000, Australia; d3.fontanarosa@qut.edu.au (D.F.);; 3 Centre for Biomedical Technologies (CBT), Queensland University of Technology, Brisbane, QLD 4000, Australia; 4 Mater Centre for Maternal and Fetal Medicine, Mater Mothers’ Hospital, South Brisbane, QLD 4101, Australia; 5 Department of Obstetrics and Gynecology, Faculty of Medicine, Universiti Malaya, Kuala Lumpur 50603, Malaysia; 6 School of Mechanical, Medical and Process Engineering and ARC Training Centre for Multiscale 3D Imaging, Modelling and Manufacturing, Queensland University of Technology, Brisbane, QLD 4000, Australia; 7 Faculty of Medicine, The University of Queensland, Herston, QLD 4006, Australia

**Keywords:** shear wave elastography, placental stiffness, placental elastography, fetal growth restriction, pre-eclampsia, ultrasound

## Abstract

**Background/Objectives**: The application of shear wave elastography (SWE) for the assessment of placental disease is still unproven and there is limited data correlating placental biomechanical properties with aberrations in fetal growth. This study investigated changes in placental shear wave velocity (SWV) in early and late fetal growth restriction (FGR). **Methods**: We analyzed three study cohorts: Pregnancies with appropriate growth for gestational age (AGA) and those with early (<32 weeks’) and late (>32 weeks’) FGR. Mean SWV at two time points was compared in the following cohorts: all FGR vs. AGA, early FGR vs. late FGR, early FGR vs. AGA, and late FGR vs. AGA. **Results**: The study comprised 222 women—79 (35.6%) FGR and 143 (64.4%) AGA. Of the FGR pregnancies, 37 (46.8%) were early and 42 (53.2%) were late. On multivariate analysis mean, SWV was not increased in FGR compared to AGA placentae (β 0.21, 95% CI −0.17–0.60, *p* 0.28). It was also not increased in early FGR compared to late FGR or AGA placentae (β 0.36, 95% CI −0.06–0.77, *p* 0.09). We observed an effect measure modification by pre-eclampsia, increasing mean SWV to a greater extent in AGA compared to FGR cases. **Conclusions**: Although previous studies have shown an association between placental SWV and FGR, our study showed no difference between cases and controls. The interaction of pre-eclampsia indicated that SWE may have a greater role in pre-eclampsia than in FGR alone. Further investigation of the influence of increased maternal vascular pressure on placental stiffness would be beneficial.

## 1. Introduction

Fetal growth restriction (FGR) is a common complication of pregnancy, and a leading cause of stillbirth and perinatal morbidity and mortality worldwide [1,2]. Growth restricted neonates are also at disproportionately increased risk of developing chronic non-communicable diseases throughout their lifetime [3,4]. Perturbations in utero-placental circulation and placental dysfunction are key factors in the etiology of FGR and pre-eclampsia [5]. Placentae from pregnancies complicated by FGR show characteristic macro and microscopic histopathological abnormalities, such as ischemic and haemorrhagic infarctions, fibrosis and parenchymal hemorrhage, which are reflective of maternal vascular malperfusion and hypoxia-induced insult to the placental villous tree [5,6,7]. Although these changes are readily appreciated after birth, prenatal ultrasound assessment of placental morphology is poorly correlated with these findings [8,9]. Instead, evidence of placental dysfunction is inferred from the presence of a small-for-gestational-age (SGA) fetus, decrease in fetal growth velocity and/or characteristic fetoplacental Doppler changes [4,10,11,12].

Shear wave elastography (SWE) is an emerging ultrasound technology that has shown some promise in measuring tissue elasticity [13,14,15]. This technique involves the transmission of a modified sound wave of greater intensity than conventional diagnostic ultrasound, resulting in lateral deformation of tissue. As the mechanical properties of biological tissues are controlled by their viscoelasticity, these elastography techniques can be utilized to quantify changes in the “stiffness” of biological tissue which are altered during inflammation and fibrosis, and may also help differentiate between healthy and impaired tissue [15,16,17]. The measured velocity of the shear wave propagating away from the pulse is proportionate to the rigidity of the tissue, with higher mean shear wave velocity (SWV) observed in stiffer and more dense tissues [17]. SWE as a non-invasive and reproducible marker of tissue inflammation and disease can be used to assess the severity of fibrosis and inflammation in various tissues [13,14,17,18] and this technique has recently been applied to assessing the biomechanical properties of placentae, with variable results [14,16,19]. Although various investigators have attempted to correlate placental stiffness measured by elastography with placental function in pregnancies with FGR, the evidence supporting its reliability and utility as a proxy for placental function is limited and generally inconclusive [20,21,22,23,24,25,26,27].

The aim of this study was to prospectively investigate whether, compared to controls, shear wave velocity is increased in placentae from pregnancies with confirmed early and late FGR defined using the International Society for Ultrasound in Obstetrics and Gynecology’s (ISUOG) Delphi consensus diagnostic criteria [28].

## 2. Materials and Methods

### 2.1. Design and Study Population

This was a prospective, observational, longitudinal study of women with a singleton fetus referred to the Mater Centre for Maternal and Fetal Medicine at the Mater Mothers’ Hospital in Brisbane, Australia between May 2022 and May 2023. All women provided informed consent for involvement in the study. Cases with known chromosomal or genetic abnormalities or major structural malformations were excluded. The study was conducted in accordance with the Declaration of Helsinki [29], with ethical and governance approvals provided on 9 September 2020 by the Mater Misericordiae Limited Human Research Ethics Committee (HREC/MML/66263) and the Mater Governance Office, respectively. Administrative review approval was also provided on 2 February 2024 by the Queensland University of Technology Office of Research Ethics and Integrity (HE-AdRev 2024-8281-17583). Maternal demographic data, relevant medical and obstetric history, and biometric data were collected.

Early and late onset FGR were defined using the ISUOG Delphi consensus criteria, defined as follows: Early FGR (<32 + 0 weeks): (1) Abdominal Circumference (AC) or Estimated Fetal Weight (EFW) < 3rd centile, (2) absent end-diastolic flow in umbilical artery, or (3) AC or EFW < 10th centile in combination with Uterine Artery Pulsatility Index (UtA PI) > 95th centile and/or Umbilical Artery Pulsatility Index (UA PI) > 95th centile. Late FGR (≥32 + 0 weeks): (1) AC or EFW < 3rd centile, (2) at least two out of three of the following: AC or EFW < 10th centile, AC or EFW crossing more than two growth quartiles and Cerebroplacental Ratio (CPR) < 5th centile or UA PI > 95th centile. A fetus appropriate for gestational age (AGA) was defined as one with AC and EFW > 10th centile and UA PI < 95th centile for gestational age [11,28].

The referent population was women with fetuses that were AGA. All fetal growth and wellbeing ultrasound and shear wave elastography examinations were performed by a single sonographer (EC) consistent with ISUOG practice guidelines [11,30] at 1–4-week intervals until birth, determined by the severity of FGR. The EFW was calculated using Hadlock’s formula [31]. At all ultrasound scans, the UA PI, Middle Cerebral Artery Pulsatility Index (MCA PI) and Ductus Venosus Pulsatility Index (DV PI) and Ductus Venosus Peak Velocity Index for Veins (DV PVIV) were measured and referenced against appropriate gestational charts [32,33,34]. The CPR was calculated as the ratio of the MCA PI and UA PI.

### 2.2. Equipment and Methods

**Figure 1:**
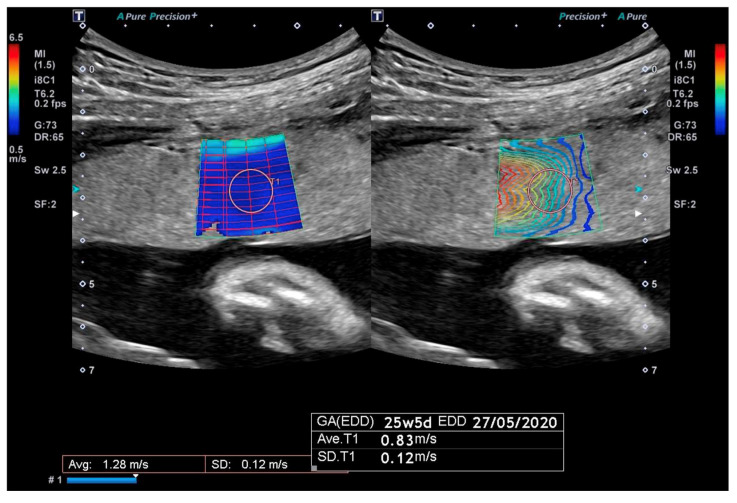
Placental 2D-SWE display demonstrating acquisition of SWV measurement with 10 mm region-of-interest (ROI); left image shows 2D color-coded shear wave speed map; right image shows reliability propagation map with parallel lines representing areas of high reliability.

SWE measurements were obtained using a Canon Aplio i-series 600 ultrasound system (Canon Medical, Otawara-shi, Japan) with a curved 8C1 transducer, using the method described in our previous manuscripts [35,36]. Summarily, the placenta was examined in gray-scale imaging to determine the shortest distance from transducer to placenta whilst maintaining a plane roughly perpendicular to the placental mass. As shown in Figure 1, the SWE acquisition box was positioned centrally within the placental mass, in an area of parenchymal homogeneity. SWV measurements were taken using the system’s single-shot technique, discharging a 2.5 MHz acoustic radiation force pulse over a single frame. A 5 mm region of interest (ROI) is placed in an area of uniform reliability as indicated by the system’s propagation map. The mean SWV (m/s^2^) was derived from thirteen separate ROIs within the same SWE acquisition box [37]. Mean SWV where the standard deviation of the mean was greater than 10 percent were excluded as this was indicative of unsatisfactory reliability of the measurement [38,39]. If thirteen measurements of reliable quality could not be achieved due to suboptimal SWV acquisition, the assessment was considered inadequate and the data excluded from the study. As the depth of ROI is known to affect measurement reliability, the average depth of the thirteen ROIs was also calculated and integrated into the analysis [38]. The duration of the elastography examination did not exceed five minutes per examination and care was taken to ensure no fetal tissue was included in the SWE acquisition [40].

All data (maternal demographic information, ultrasound measurements and other relevant clinical details) were collected and recorded using REDCap™ (Research Electronic Data Capture, Version 15.5.0). REDCap™ is a secure, web-based software platform designed to support data capture for research projects [41,42]. All healthcare providers were blinded to the resultant SWV data, and any clinical management decisions were made primarily on the findings of fetal biometry, EFW, fetal Dopplers, and/or the overall maternal clinical condition.

Participants were categorized into early FGR, late FGR and AGA groups according to the Delphi consensus [28]. We analyzed ultrasound and SWE data from the last examination prior to 32 weeks’ gestation and the last examination prior to delivery.

We compared SWV at the two time points for the following cohorts:

- All FGR vs. AGA
- Early FGR vs. late FGR
- Early FGR vs. AGA
- Late FGR vs. AGA

Clinically relevant confounders [43,44] of the association between FGR and z-score of SWV included body mass index (BMI) and tissue depth [45]. Pre-eclampsia was considered both a confounder and an effect measure modifier because it is associated with FGR and SWV. As missing data were less than 1%, a complete case analysis was used. The primary study outcome was the z-score of mean SWV.

### 2.3. Statistical Analysis

Preliminary assessment of the associations between FGR status, health and demographic characteristics, and ultrasound findings were performed using chi-squared tests for categorical variables and Analysis of Variance (ANOVA) or Kruskal–Wallis ANOVA according to distribution. The distribution of continuous variables was evaluated using histograms and mean/standard deviation < 2 to indicate extreme skewness [46]. Preliminary assessment of the associations between FGR, early-onset FGR, late-onset FGR, pre-eclampsia, and SWV were performed using box plots. Multivariable generalized linear models were built, using a Gaussian distribution and identity link, adjusting for clinically relevant confounders. The correlation between average tissue depth and BMI was determined using Spearman’s rank correlation coefficients, with correlations considered significant at *p* < 0.05. Tissue depth was omitted from all models of the last scan before 32 weeks due to collinearity with BMI (*p* < 0.0001 for all models). The assumption of normality was checked by plotting histograms of deviance residuals. The assumption of homoskedasticity was checked by plotting deviance residuals against linear fitted values. Cook’s distance was used to investigate observations with undue influence.

As this was an exploratory study and previous studies investigating the association between FGR and SWV are limited, sample size calculations were not performed. Significance was set at α = 0.05 for all statistical tests. The reporting of this study conforms to the STROBE (Strengthening the Reporting of Observational Studies in Epidemiology) statement [47]. Statistical analyses were performed using Stata 18.5^®^ (Statacorp LLC, College Station, TX, USA).

## 3. Results

**Figure 2:**
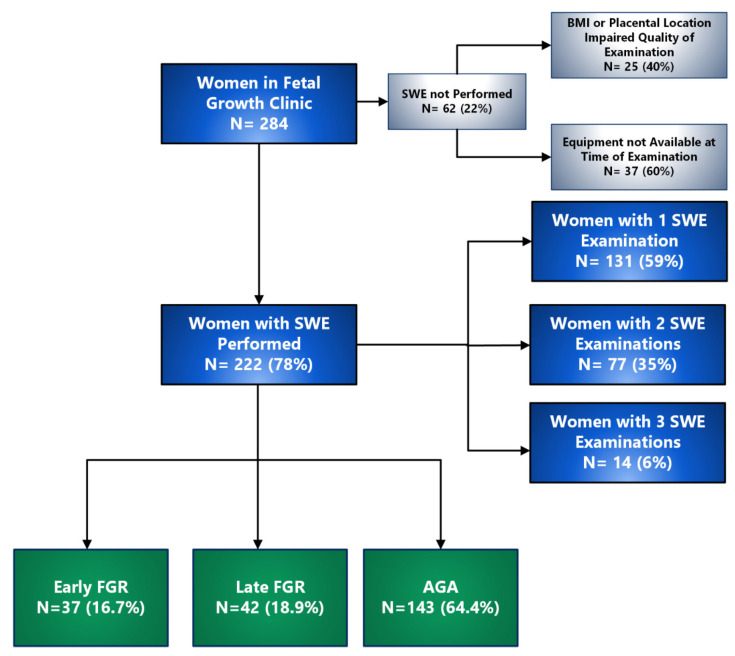
Study flow chart. SWE: shear wave elastography, BMI: body mass index, FGR: fetal growth restriction, AGA: appropriate for gestational age.

Over the study period, 247 women met the inclusion criteria and agreed to participate. SWE was unable to be performed in 25 women (8.8%) because increased maternal BMI or posterior placental location impaired the quality of the examination. The final cohort comprised 222 women (89.9%) in whom SWV was successfully measured (Figure 2). Of these, 35.6% (79/222) were FGR and 64.4% (143/222) were AGA. Of the FGR cases, 46.8% (37/79) were early FGR, and 53.2% (42/79) were late FGR. Table 1 outlines the demographic and health characteristics of the study population. Women with FGR were more likely to smoke, have diabetes mellitus and gestational hypertension and/or pre-eclampsia.

**Figure 3:**
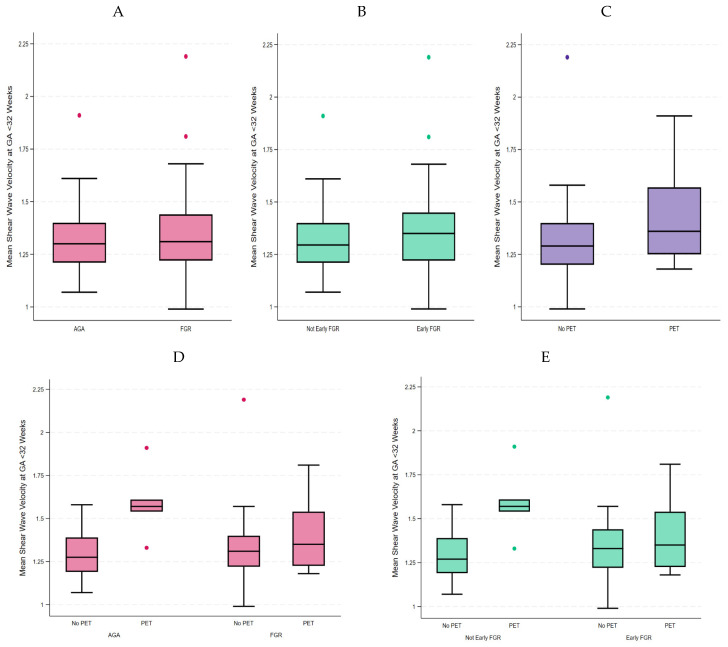
Mean SWV at final scan before 32 weeks: (**A**) All FGR, (**B**) Early FGR, (**C**) Pre-eclampsia, (**D**) FGR stratified by pre-eclampsia, and (**E**) Early FGR stratified by pre-eclampsia. GA: gestational age, AGA: appropriate for gestational age, FGR: fetal growth restriction, PET: pre-eclampsia.

When we compared the last examination < 32 weeks’ gestation, we found on univariate analysis that the z-score of mean SWV was not significantly different in any FGR vs. AGA placentae. It was also not significantly different between placentae from early FGR vs. late FGR, early FGR vs. AGA, or late FGR vs. AGA cases. Table 2 lists the univariate analysis results. On multivariate analysis, the mean SWV was not increased in FGR compared to AGA placentae (β 0.21, 95% CI −0.17–0.60, *p* 0.28). It was also not increased in early FGR compared to late FGR or AGA placentae (β 0.36, 95% CI −0.06–0.77, *p* 0.09), as demonstrated in Figure 3A,B. There was an effect measure modification by pre-eclampsia which increased the mean SWV to a greater extent in AGA compared to any FGR or early FGR cases (Figure 3C–E).

**Figure 4:**
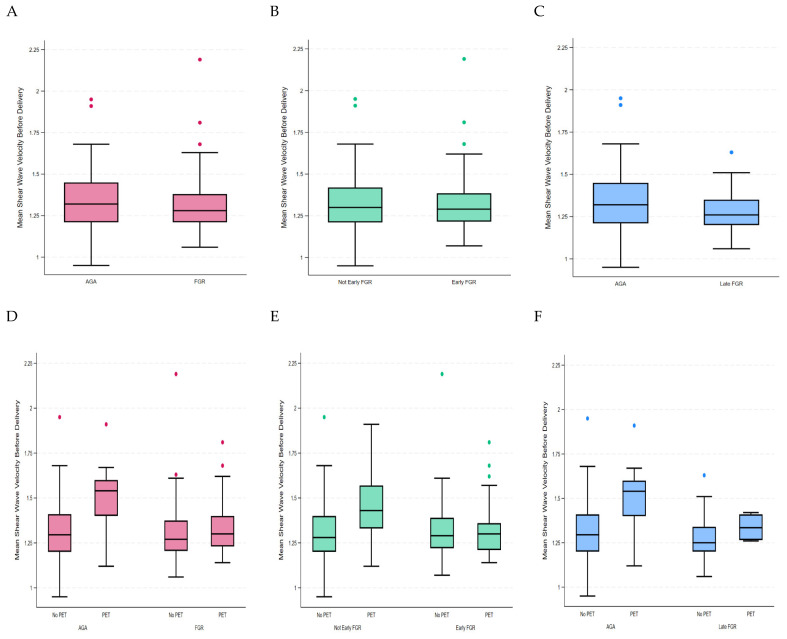
Mean Shear Wave Velocity at final scan before birth: (**A**) All FGR, (**B**) Early FGR, (**C**) Pre-eclampsia, (**D**) FGR, (**E**) Early FGR, and (**F**) Late FGR stratified by pre-eclampsia. AGA: appropriate for gestational age, FGR: fetal growth restriction, PET: pre-eclampsia.

When the last examination prior to birth was assessed, we found that the z-score of mean SWV was not increased in FGR compared to AGA placentae (−0.09, 95% CI −0.36–0.18 *p* < 0.001). There was also no increase in the z-score between early FGR (0.09, 95% CI −0.22–0.39 *p* < 0.001) or late FGR (−0.29, 95% CI −0.65–0.07 *p* < 0.001) vs. AGA placentae. On multivariate analysis, as demonstrated in Table 3, the mean SWV was not increased in FGR vs. AGA placentae (β −0.002, 95% CI −0.28–0.28, *p* 0.99). The mean SWV was also not increased in early FGR compared to AGA placentae (β 0.13, 95% CI −0.21–0.46, *p* 0.46). Similarly, the mean SWV was not increased in late FGR compared to AGA placentae (β −0.13 95% CI −0.48–0.22, *p* 0.47). This is shown in Figure 4A–C. A similar association and effect measure modification due to pre-eclampsia was again present (Figure 4D–F).

## 4. Discussion

In this study of well characterized FGR cases, we did not find any difference in placental SWV between FGR cases and AGA controls. We also found no difference in SWV between early FGR vs. late FGR placentae or early FGR and late FGR placentae compared to AGA placentae. Our findings suggest that evaluation of placental SWV as a surrogate measure of placental function may not be useful in assessing either the severity or progression of placental dysfunction in pregnancies complicated by early or late FGR. We undertook the current study because in a previous SWE study of placentae from SGA pregnancies compared to AGA controls [36] we found no difference in mean SWV between cases and control. We postulated that the severity of placental dysfunction in the SGA cohort may not have been substantial enough to cause a change in placental mechanical properties which, in turn, would be reflected by differences in SWV. Therefore, in this prospective study, we specifically included cases of early and late FGR and compared them to AGA controls. Our results also indicate that pre-eclampsia in isolation, rather than FGR, has a greater impact on placental SWV.

A strength of our analysis is the reliability of the SWV measurements we obtained. Previous studies have shown that measurement of SWV is highly influenced by depth of the ROI, attenuation of overlying tissues, or operator-related variations [38,39]. In our study, all measurements were performed by a single experienced sonographer according to a strict imaging protocol. A previous publication from our group [38] concluded that accurate assessment of mean SWV requires a large number of small ROIs when assessing a large inhomogeneous structure such as the placenta. Our study derived the mean SWV from thirteen 5 mm ROIs, and our threshold for remeasurement or rejection of the sample limited the standard deviation of mean SWV to 10 percent. Our study’s large number of observations and rigorous measurement protocols ensured a high level of reliability and reproducibility. This is a significant consideration as SWE reliability studies have shown a wide variation in results and high operator dependency [15,39,48]. Comparable placental elastography studies have demonstrated a wide variation in SWV measurements of up to 40 percent [20,22,23,24,49]. Increased maternal BMI has previously been shown to influence mean SWV [45]. Our analysis adjusted for maternal BMI, however the independent increased risk of pre-eclampsia in high BMI women may compound this effect [50].

We acknowledge that there are some limitations of this study, the first being that it was conducted in a single center. The second limitation is that subjects were recruited when initially referred to Maternal Fetal Medicine for a medically indicated growth scan, and then followed up with a subsequent scan as clinically required. This resulted in data being collected at variable gestational ages; thus, data collection points were not uniform. Furthermore, it is possible that some women who were referred after 32 weeks’ gestation may have had undiagnosed early FGR.

Deficiencies in placental angiogenesis underpin the etiology of both FGR and pre-eclampsia [5,7,12,51]. Failure of the uterine spiral arteries to remodel during early pregnancy gives rise to hypoperfusion of the placenta and suboptimal maternal-fetal exchange, with both growth restriction and pre-eclampsia being common outcomes [5]. Many previous investigations of placental SWV in pregnancies with FGR and/or pre-eclampsia have assumed that there is relative homogeneity in placental biomechanical properties. However, the placenta is a highly complex vascular organ representing the interface between maternal and fetal circulations and has a wide phenotype [52]. In their review of the biomechanical properties of the umbilical-placenta system, Saw et al. (2021) [48] suggest that the pressure and stress of blood flow within placental tissue could potentially distend and alter placental parenchyma. Flow conditions in the placenta in vivo reflect both the maternal (uterine artery) and fetal (umbilical artery) vascular resistance, and vascular pressure applied to placental tissue from high-resistance uterine artery flow (such as in pre-eclampsia) may extrinsically influence vascular elasticity within the organ [53,54,55,56]. In this way, biofluid mechanical properties may also influence the stiffness of placental tissue [18,48]. This observation may possibly account for our finding of increased SWV in pre-eclampsia, both with and without FGR, and thus SWE may have more of a role to play in pre-eclampsia than in FGR alone. Future work should include investigation of the influence of increased maternal vascular pressure on placental stiffness in the setting maternal hypertension in pregnancy.

## 5. Conclusions

The complexities in diagnosing and monitoring placenta-mediated early and late fetal growth restriction remain a major challenge for clinicians. Current obstetric practice relies on ultrasound-derived biometric and Doppler measurements as inferred measures of placental function. Ultrasound elastographic measurement of the biomechanical properties of placental tissue has offered some promise in supporting the diagnosis of placenta-related growth disruption. However its utility for placental assessment remains to be confirmed, and although previous research has shown some correlation between increased placental stiffness and fetal growth disturbance [20,21,22,23,24,25,26,27], our results did not support this finding. The outcomes from our study show potentially limited utility for SWE in the evaluation of FGR placentae.

## Figures and Tables

**Table 1:** Demographic table, ultrasound parameters, and pregnancy outcomes.

	Total	Early FGR	Late FGR	AGA	*p*-Value
	N = 222	N = 56	N = 33	N = 133	
Age	31 ± 6	33 ± 5	30 ± 5	31 ± 6	0.058
Body Mass Index at recruitment (kg/m^2^)	25 (21, 31)	26 (23, 31)	24 (21, 27)	24 (21, 31)	0.062
Mean Arterial Pressure at time of recruitment (mmHg)	82 (77, 88)	83 (77, 93)	82 (78, 87)	82 (77, 87)	0.190
Nulliparous	86 (38.7%)	24 (42.9%)	14 (42.4%)	48 (36.1%)	0.610
Born in Australia	94 (42.3%)	21(37.5%	13 (39.4%)	60 (45.1%)	0.890
Born outside Australia	128 (57.7%)	35 (62.5%)	20 (60.6%)	73 (55.9%)	0.720
Mode of Conception					0.066
Spontaneous	205 (92.3%)	48 (85.7%)	30 (90.9%)	127 (95.5%)	
Assisted Reproductive Technology	17 (7.7%)	8 (14.3%)	3 (9.1%)	6 (4.5%)	
Smoking during pregnancy	21 (9.5%)	5 (8.9%)	7 (21.2%)	9 (6.8%)	0.039
Alcohol during pregnancy	3 (1.4%)	2 (3.6%)	0 (0.0%)	1 (0.8%)	0.240
History of SGA baby	73 (32.9%)	22 (39.3%)	11 (33.3%)	40 (30.1%)	0.470
Previous stillbirth	15 (6.8%)	5 (8.9%)	0 (0.0%)	10 (7.5%)	0.230
Previous preterm birth	27 (12.2%)	12 (21.4%)	4 (12.1%)	11 (8.3%)	0.041
Hypertension in current pregnancy	43 (19.4%)	18 (32.1%)	4 (12.1%)	21 (15.8%)	0.018
Pre-eclampsia in current pregnancy	36 (16.2%)	21 (37.5%)	4 (12.1%)	11 (8.3%)	<0.001
Low dose aspirin use	62 (27.9%)	22 (39.3%)	3 (9.1%)	37 (27.8%)	0.009
Connective tissue disease in pregnancy	4 (1.8%)	3 (5.4%)	0 (0.0%)	1 (0.8%)	0.066
Low Molecular Weight Heparin use	12 (5.4%)	7 (12.5%)	0 (0.0%)	5 (3.8%)	0.020
Diabetes in pregnancy	65 (29.3%)	25 (44.6%)	5 (15.2%)	35 (26.3%)	0.006
Renal disease in pregnancy	3 (1.4%)	1 (1.8%)	0 (0.0%)	2 (1.5%)	0.76
Gestational age at last scan before 32 weeks’ gestation	28 (26, 30)	28 (26, 30)	31 (30, 31)	28 (25, 30)	0.021
Gestational age at last scan before birth	35 (32, 36)	33 (30, 35)	36 (35, 37)	36 (32, 36)	<0.001
EFW at last scan before 32 weeks’ gestation	1126 (764, 1418)	844 (628, 1223)	1420 (1252, 1480)	1171 (776, 1485)	0.003
EFW at last scan before birth	2190 (1627, 2493)	1571 (1106, 1968)	2136 (1895, 2288)	2371 (1964, 2622)	<0.001
Gestational age at birth	38 (37, 39)	37 (32, 37)	37 (37, 38)	38 (37, 39)	<0.001
Mean SWV at last scan before 32 weeks’ gestation (m/s^2^)	1.30 (1.22, 1.41)	1.35 (1.22, 1.45)	1.23 (1.19, 1.32)	1.30 (1.21, 1.40)	0.330
Mean SWV at last scan before delivery (m/s^2^)	1.29 (1.21, 1.41)	1.29 (1.22, 1.38)	1.26 (1.20, 1.35)	1.32 (1.21, 1.45)	0.360
Average depth of SWV sample at last scan before 32 weeks’ gestation (cm)	4.78 (3.82, 5.51)	4.54 (3.65, 5.72)	4.88 (4.26, 5.14)	4.90 (3.87, 5.60)	0.640
Average depth of SWV sample at last scan before delivery (cm)	4.48 (3.80, 5.22)	4.30 (3.68, 5.02)	4.20 (3.72, 5.00)	4.67 (3.90, 5.42)	0.280

Values represent N(%) for categorical variables and mean (±standard deviation) or median (25%, 75%) for continuous variables, according to distribution. FGR: fetal growth restriction, AGA: appropriate for gestational age, SGA: small for gestational age, EFW: estimated fetal weight, SWV: shear wave velocity.

**Table 2:** Univariable analysis. FGR: fetal growth restriction, AGA: appropriate for gestational age, CI: confidence interval.

Model 1	FGR vs. AGA (Last Scan Before 32-Weeks’ Gestation)	
	Univariable Coefficient (95% CI)	*p*-Value
FGR	0.13 (−0.25, 0.51)	0.51
**Model 2**	Early FGR vs. No early FGR (last scan before 32 weeks’ gestation)	
	Univariable coefficient (95% CI)	*p*-value
Early FGR	0.27 (−0.13, 0.66)	0.18
**Model 3**	FGR vs. AGA (last scan before delivery)	
	Univariable coefficient (95% CI)	*p*-value
FGR	−0.09 (−0.36, 0.18)	0.52
**Model 4**	Early FGR vs. AGA (last scan before delivery)	
	Univariable coefficient (95% CI)	*p*-value
Early FGR	0.09 (−0.22, 0.39)	0.57
**Model 5**	Late FGR vs. AGA (last scan before delivery)	
	Univariable coefficient (95% CI)	*p*-value
Late FGR	−0.29 (−0.65, 0.07)	0.12

**Table 3:** Multivariable analysis adjusting for pre-eclampsia and body mass index and considering the interaction (|) between fetal growth restriction and pre-eclampsia.

Model 1	FGR vs. AGA (Last Scan Before 32-Weeks’ Gestation)	
Variable	Multivariable Coefficient (95% CI)	*p*-Value
FGR	0.21 (−0.17, 0.60)	0.28
Pre-eclampsia	1.48 (0.69, 2.27)	<0.001
FGR # Pre-eclampsia	−1.37 (−2.32, −0.42)	0.005
BMI	0.06 (0.03, 0.08)	<0.001
**Model 2**	Early FGR vs. No early FGR (last scan before 32 weeks’ gestation)	
Variable	Multivariable coefficient (95% CI)	*p*-value
Early FGR	0.36 (−0.06, 0.77)	0.09
Pre-eclampsia	1.50 (0.72, 2.28)	<0.001
FGR # Pre-eclampsia	−1.52 (−2.47, −0.56)	0.002
BMI	0.06 (0.03, 0.08)	<0.001
**Model 3**	FGR vs. AGA (last scan before delivery)	
Variable	Multivariable coefficient (95% CI)	*p*-value
FGR	−0.002 (−0.28, 0.28)	0.99
Pre-eclampsia	1.10 (0.54, 1.66)	<0.001
FGR # Pre-eclampsia	−1.09 (−1.80, −0.38)	0.003
BMI	0.05 (0.03, 0.07)	<0.001
**Model 4**	Early FGR vs. AGA (last scan before delivery)	
Variable	Multivariable coefficient (95% CI)	*p*-value
Early FGR	0.13 (−0.21, 0.46)	0.46
Pre-eclampsia	0.90 (0.41, 1.39)	<0.001
FGR # Pre-eclampsia	−1.06 (−1.76, −0.36)	0.003
BMI	0.05 (0.03, 0.07)	<0.001
**Model 5**	Late FGR vs. AGA (last scan before delivery)	
Variable	Multivariable coefficient (95% CI)	*p*-value
Late FGR	−0.13 (−0.48, 0.22)	0.47
Pre-eclampsia	1.10 (0.57, 1.63)	<0.001
FGR # Pre-eclampsia	−0.71 (−1.74, 0.33)	0.18
BMI	0.05 (0.03, 0.07)	<0.001

FGR: fetal growth restriction, AGA: appropriate for gestational age, BMI: body mass index, CI: confidence interval, #: interaction with.

## Data Availability

Data used to produce the results in this article will be available to any researcher, provided appropriate ethics approval, inter-institutional data sharing agreements, and other regulatory requirements are in place.

## Abbreviations

The following abbreviations are used in this manuscript:

SWE	Shear wave elastography
SWV	Shear wave velocity
FGR	Fetal growth restriction
AGA	Appropriate (growth) for gestational age
ISUOG	International Society for Ultrasound in Obstetrics and Gynecology
AC	Abdominal circumference
EFW	Estimated fetal weight
CPR	Cerebroplacental ratio
UA PI	Umbilical artery pulsatility index
MCA PI	Middle cerebral artery pulsatility index
DV PI	Ductus venosus pulsatility index
DV PVIV	Ductus venosus peak velocity index for veins
ROI	Region of interest
BMI	Body mass index
ANOVA	Analysis of Variance
SGA	Small for gestational age
CI	Confidence interval
PET	Pre-eclampsia toxemia
